# Latent Medication*Read before American Institute, at Milwaukee, 1880.

**Published:** 1882-03

**Authors:** P. P. Wells

**Affiliations:** Brooklyn, N. Y.


### HOMŒOPATHIC PHYSICIAN.

A MONTHLY JOURNAL OF MEDICAL SCIENCE.

"If our school ever gives up the strict inductive method of Hahnemann, we are lost, and deserve only to be mentioned as a caricature, in the history of medicine."—CONSTANTINE HERING.

Vol. II. MARCH, 1882. No. 3.

### LATENT MEDICATION.*

P. P. WELLS, M. D., BROOKLYN, N. Y.

"The original sin is not being able to wait for a thing."—*W. Gross.*

Whether, indeed, impatience be really the parent of *all* practical sins in homœopathic prescribing, may, perhaps, be questioned. But it may not be questioned that it is one of the most frequent causes of mistake and disappointment. It is so natural to be in haste to realize the fruits of our expectations, that waiting for these is often more than a weariness, and if these be long delayed, the disposition to do something more or different, hoping for a better or more speedy result, is often quite irresistible. If we have made our selection of the remedy already given, with due reference to the requirements of the law of cure, this temptation to a too early change of the remedy, or to add others to it, in the hope of a better result, should be resisted with the utmost firmness from the beginning. To be *sure* of the right selection of the remedy first given is the one great and first duty of the prescriber; the second is, when he has given this, to let it alone, that it may accomplish its work without interference from whatever of anxiety on the part of the prescriber, or the presence of other drugs or appliances which may modify or destroy its legitimate action. To fortify this firmness in the beginning, let him remember this, which his seniors ought to have learned long ago, that for the

* Read before American Institute, at Milwaukee, 1880.

development of visible curative action of drugs given to the sick, *time* is required; and this, further, that different drugs vary greatly in the length of the period necessary for this; with some it is minutes, with others, hours, days, or weeks even. And this, further, that till the lapse of this required period, nothing of good can come to the patient by the substitution or addition of other drugs to that already given, this being the specific for the case in hand. That it is so, we here take for granted. For failing to be this, rules for its administration are useless; indeed, they are impossible. And then, for the further strengthening of the timid, let it be borne in mind, that as with drugs, so with diseases. These often demand patience before they yield to the curative impression of the drug; they will have time before they respond in ease or convalescence. That different forms of disease, and often different examples of the same disease, require different periods: some only a brief, others a longer time. And these periods of time during which we are looking anxiously for visible improvement, often long delayed, are the necessary part of the experience of a cure. And this further, and chiefly, that during this period, the drug given, being the specific, is not necessarily inactive because results of its action are not yet externally visible. This is the period of *latent medication.* It is a necessary part of every true homœopathic cure. It is often limited to a few moments, while at other times it is extended to days. The duration of this period is determined by the nature of the morbific cause and the profoundness of the impression it has made on the organism, on the one hand, and on the other by the susceptibility of the organism to medicinal impression.

In cases of croup, for example, relief is often found at the expiration of a few minutes after the dose has been given; in typhoid fever, on the other hand, not until after days. During these days of anxious watching, the action of the drug, though invisible as to any external signs of recovery, is nevertheless potent in affecting the process of cure. The susceptibilities of the organism are so far depressed by the impact of the morbific cause that they only respond in visible improvement after repeated impress of the curative. These repeated impressions are not fruitless in the process of cure, because traces of their activity are invisible. They are expended, for the time, in recovering the depressed forces from the power of the morbific cause, till bye and bye they may be able to effect the desired visible manifestations of returning health.

The remedy, being the true specific, is from the beginning actively operative in bringing about the result it is its nature to accomplish, and, therefore, the necessity of obedience to the rule which nature and law have established—that it be let alone to accomplish the necessary part of the process of cure. It is for no conceivable reason or pretence to be interfered with by the interposition of other drugs, or resort to whatever of suggestion from our own anxieties or those of others. It being the specific, it is, therefore, the best possible remedy for the case; and hence, to substitute another for it, is to give one which is something less than best. To interpose another with this is to endanger the legitimate action of the specific, subjecting it, it may be, either to total destruction, or to such a modification of its true effects as may seriously embarrass the future treatment of the case, and perhaps even to convert a case which is curable, it being left to the undisturbed action of its specific, to one, after this pernicious interference, wholly beyond the control of remedies, selected by whatever of skill. If the specific be the best remedy and it fails, then that which is no specific, and, therefore, not so good, cannot bring us success. It and all others will necessarily fail.

If these views are correct, then it follows that mere delay in the appearance of visible curative effects is not a valid reason for changing the selected remedy in a case under treatment. It may be the best of reasons for revising the choice of our remedy, but never for its change, unless another which is *more like* is found.

This fact of latent medication was well seen in a case of typhoid fever, which was treated by the writer many years ago. The patient was a young man, 20 years of age, a clerk, of country birth, having been a resident of Brooklyn but a few months. He was first seen and prescribed for by the writer of this, the third day after the attack had confined him to his boarding-house. Previous to his illness he had always been robust, enjoying perfect health. He was found perfectly deaf and in a muttering delirium, talking constantly to himself, not at all noticing his surroundings, or giving the slightest heed to endeavors to arouse him to answer questions. He was in his imaginary world, quietly interested in it, busy with its images, while he was wholly separated from and lost to all that was real about him. He was sleepless, but quiet except he made constant efforts to reach and grasp imaginary objects in the air There was a constant jerking of the tendons of both upper and lower extremities; his evacuations were passed into his bed involuntarily and unnoticed. He had frequent brown, liquid, offensive discharges from the bowels. The tongue was dry, hard and brown. The lips dry and with the gums covered with dark sordes. Skin hot and dry, or at times there was a slight hot perspiration.

This was the condition of the patient as early as the third day of the attack. I had never before seen so formidable an array of symptoms, developed so early in any case of this fever. The prognosis was, of course, unfavorable. It was the practice of the writer, at this early period of his homœopathic experience, to carry his materia medica with him in his daily rounds to the sick; and at the bedside of the patient, after getting his symptoms as clearly and completely as possible, there to study it, that he might find, if possible, the "*most like*" which cures. He thus visited and studied this case of fever and the materia medica, twice each day for fourteen days. His first study was a careful and protracted one, and the remedy was selected only after each drug which in its record carried symptoms similar to those of the case, had been examined. That one was chosen which had most of the symptoms and there, in its record, the most strongly expressed, that is, which was the "*most like.*" This was given with the clear conviction that the *best* chance for the life of the patient was in this one drug, and that this was but a poor one. In the evening, after the first visit, he was "*no better.*" The study was repeated, and the result was the choice of the same drug as in the morning. It was still the "*most like.*" So it was the next morning, and the next evening, and so each day for fourteen days, the same sad disappointing "*no better.*" The same earnest study resulting in the same choice of remedy. It was in the earlier days of the treatment of this case, after repeated sadness because there was no amendment, and therefore repeated temptations to try some other drug, that this temptation was overcome by the thought that if this remedy which is the "*most like,*" and therefore the best, fails, what reason is there to hope for better results from that which is less "*like,*" and therefore not so good? It was this that, through that succession of heart-aching studies, held the prescriber to the one chosen remedy, though it brought no visible improvement for so many days. The result justified both the choice of the remedy and the adherence to it, through this protracted period of what perhaps cannot be better named than "*Latent Medication.*" That there was such a fact was not then recognized. That there *is*, all enlightened practical experience will now testify. On the fourteenth day the whole train of symptoms cleared away, and in so remarkable a manner as to suggest the passing of a cloud before the sun. The convalescenee was brief and perfect. The whole case has been a useful lesson to its attending physician; illustrating and enforcing the value of our fundamental rule for the right administration of our law—the right selection of the remedy, and then adherence to it through whatever of time or discouragement, till it ceases to be the one "*most like*" to the symptoms we are treating.

The disastrous effects of a violation of this rule by a change of remedy for any other reason than greater similarity, is illustrated by the following case, also of typhoid fever: The patient was a man 50 years of age, of robust frame, who had before his present attack enjoyed general good health. Two or three weeks before he sickened he buried his wife, to whom he was strongly attached. At the beginning of his illness he told his family he was going to die, and persisted in this so long as he retained consciousness. He came under my care from that of a friend, whose duties at the time called him from home. It was the tenth day of his illness. He was now in unconscious delirium, talking incessantly of dead bodies or dead persons, coffins, funerals, funeral processions, and matters related to these. He could be roused to answer questions, but immediately returned to his sad talk and fancies. He passed his evacuations involuntarily and unnoticed. He had brown, watery diarrhœa, the discharges being but slightly offensive. The tympanitis was great, the abdomen being senitive to pressure over the whole surface. The tongue dry, brown and hard. The mouth dry, and the lips and teeth covered with dark sordes. The case was carefully studied and the remedy chosen. Prognosis unfavorable, and chiefly because of the delirium, dwelling on death, dead bodies, funerals, etc. Cases of this kind had all died in the previous experience of the prescriber. Notwithstanding this unpromising state of things, the first prescription was followed by improvement in the first twenty-four hours, which continued to progress, till the delirium, involuntary discharges, tympanitis, dry tongue and sordes had disappeared, and the case was promising recovery from all these untoward symptoms, and a break in my uniform experience of fatal termination of such cases. There was one fact only which seemed to call for special attention or anxiety—*he would not take food.* His refusal was constant and pointed. The fear of exhaustion from this cause led to a change of medicine with the hope that this might remove the one and only seeming impediment to recovery. The expedient failed. He refused food till death relieved him of its necessity. After death it was a matter of regret that the remedy first given, which had already done so much for the patient, had not been trusted for the removal of this one remaining symptom, which was ultimately fatal, notwithstanding the change. It could not be, and cannot be, otherwise than that the question should arise—was not his death due to the unsuccessful change? *

* As to the repetition of dose. I am not yet so perfectly clear, under the law, as to be to my mind certain that there are not two sides to the question, and something to be said on each. This only is clear. While improvement is progressing there should be no repetition. But in these cases of latent medication the difficulty is, that while this improvement is progressing, it is not visible. In such cases I would repeat—not too often—till improvement is visible, and then stop.

